# The Role of Water Dimers in the Initial Stage of Salt Crystallization

**DOI:** 10.34133/research.1040

**Published:** 2025-12-23

**Authors:** Jiadong Guo, Xinmeng Liu, Yunzhe Jia, Junhao Xie, Yuejian Zhang, Yipeng He, Jiyu Xu, Cui Zhang, Duanyun Cao, Sheng Meng, Ying Jiang

**Affiliations:** ^1^ International Center for Quantum Materials, School of Physics, Peking University, Beijing 100871, China.; ^2^ Interdisciplinary Institute of Light-Element Quantum Materials and Research Center for Light-Element Advanced Materials, Peking University, Beijing 100871, China.; ^3^ Beijing National Laboratory for Condensed Matter Physics, Institute of Physics, Chinese Academy of Sciences, Beijing 100190, China.; ^4^ School of Physical Sciences, University of Chinese Academy of Sciences, Beijing 100049, China.; ^5^ Beijing Key Laboratory of Environmental Science and Engineering, School of Materials Science and Engineering, Beijing Institute of Technology, Beijing 100081, China.; ^6^ Songshan Lake Materials Laboratory, Dongguan 523808, China.; ^7^ Chongqing Innovation Center, Beijing Institute of Technology, Chongqing 401120, China.; ^8^ Collaborative Innovation Center of Quantum Matter, Beijing 100871, China.; ^9^ New Cornerstone Science Laboratory, Peking University, Beijing 100871, China.

## Abstract

Probing early-stage crystallization in hydrated environments is crucial for elucidating the microscopic mechanism of crystal growth. However, capturing these processes remains challenging because of the nanometric dimensions of nanocrystals and the dynamic role of water in solvation and ion–ion interactions. Here, we employ a cryogenic scanning probe microscopy platform, which integrates qPlus-type atomic force microscopy with a frozen-solution preparation technique, to directly visualize hydrated sodium chloride (NaCl) nanocrystals at atomic resolution. We observe double- to 5-stranded ionic chain structures, which are hydrated by water dimers. Those structures promote the anisotropic growth of NaCl nanocrystals. Density functional theory calculations reveal that the water dimer can substantially stabilize the chain-shaped configurations by optimizing the water–water and water–ion interactions. In contrast, larger crystals favor isotropic crystalline lattices due to dominant bulk ionic interactions. These findings highlight the unique role of water dimers in the initial crystallization process of salts. Furthermore, this work demonstrates the potential of cryogenic scanning probe microscopy as a powerful tool for probing the crystallization process at atomic resolution, particularly in hydrated environments.

## Introduction

Crystallization in hydrated environments plays a central role in physics, chemistry, and materials science. Recent research has increasingly focused on the structure and growth behavior of nanocrystals, as their early-stage configurations critically influence the final crystal structures and properties [1–5]. However, direct observation of the initial stages of crystallization remains challenging due to the nanometric dimensions, structural heterogeneity, and complex solvation environments [6].

Recent advances in liquid-phase transmission electron microscopy (TEM), especially when combined with nanoscale confinement such as graphene liquid cells, have opened new opportunities to probe the crystallization pathways of ionic compounds at atomic resolution. For example, in the case of sodium chloride (NaCl), in situ TEM studies have revealed the formation of a transient graphite-like hexagonal intermediate phase prior to the stabilization of the rock-salt structure [7]. This provides compelling evidence for multistep nucleation pathways in confined aqueous environments. In addition, single-molecule atomic-resolution time-resolved electron microscopy has been able to capture the very moment of crystal nucleus emergence from disorder, highlighting the stochastic and transient nature of early nucleation events [8]. Despite these breakthroughs, the limited sensitivity of TEM to light elements prohibits direct visualization of hydration shells and water–ion interactions. Moreover, the nonnegligible effects of electron beam irradiation on solution chemistry and nucleation pathways remain a subject of debate [9].

On the theoretical side, molecular dynamics simulations have provided valuable insights into the microscopic mechanism of salt crystallization. For instance, simulations have demonstrated that 2-dimensional monolayer salt nanostructures can spontaneously aggregate in dilute aqueous solutions rather than dissolve, suggesting that hydrated ions can stabilize metastable nanoscale assemblies that deviate from classical dissolution–precipitation expectations [10]. More broadly, simulations suggest that depending on supersaturation, NaCl can crystallize via either 1-step direct nucleation or a 2-step pathway involving the formation of disordered, ion-rich clusters followed by structural ordering into crystalline nuclei [11–13]. Although molecular dynamics simulations have also predicted various hydrated NaCl nanocrystal structures and revealed possible pathways of water-mediated stabilization [10], none of these configurations have yet been confirmed experimentally. Consequently, the microscopic structures of hydrated NaCl nanocrystals and the precise role of water in directing crystallization pathways remain unresolved.

Scanning probe microscopy offers atomic-scale resolution on surfaces and has become a powerful tool for probing interfacial structures [14]. In particular, qPlus-based atomic force microscopy (AFM) with carbon monoxide (CO)-functionalized tips has enabled ultrahigh-resolution imaging of insulating surfaces, resolving both atomic lattices and individual chemical bonds [15]. By sensing higher-order electrostatic interactions, it can achieve submolecular resolution of water clusters in a noninvasive manner [16]. However, direct application of qPlus–AFM to crystallization processes in solution remains infeasible, primarily due to its reliance on ultrahigh vacuum and cryogenic conditions, which are incompatible with liquid-phase environments.

## Results and Discussion

In this work, we employ a homebuilt cryogenic scanning probe microscopy (cryo-SPM) platform, which integrates a frozen-solution preparation apparatus with a qPlus–AFM system. This setup enables direct transfer of frozen solutions in glassy states onto various substrates under contamination-free conditions, allowing atomic-resolution characterization of frozen solutions. We investigate aqueous NaCl solution, chosen for its high solubility and simple rock-salt crystal structure, which make it ideal for studying the fundamental processes of crystallization in solution. We are able to directly visualize the atomic structures of hydrated NaCl nanocrystals and reveal the critical role of water dimers in the anisotropic growth of NaCl nanocrystals.

**Fig. 1.**
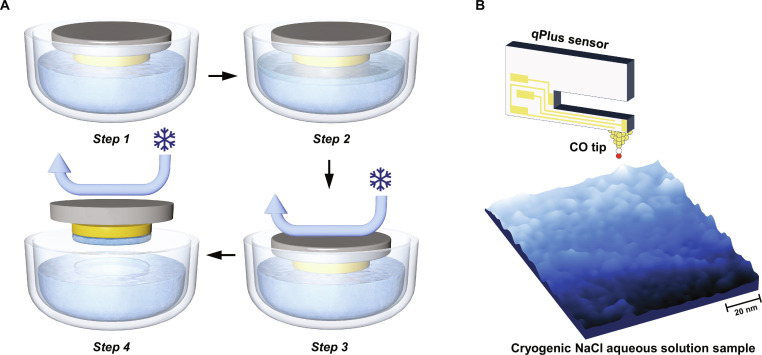
Schematic of the experimental setup. (A) Schematic of the cryogenic scanning probe microscopy (cryo-SPM) sample preparation procedure. The room-temperature substrate is brought into contact with the frozen-solution surface (step 1). Upon contact, the frozen-solution melts, forming a thin interfacial liquid layer (step 2). A continuous flow of liquid nitrogen (LN_2_) is introduced to rapidly freeze both the melted solution and the substrate together (step 3). The frozen solution is cleaved and transferred onto the substrate by detaching the substrate from the frozen solution (step 4). (B) Constant-frequency-shift atomic force microscopy (AFM) image of a cryogenic NaCl aqueous solution sample after annealing at 160 K (frequency shift set point: −200 mHz; image size: 100 nm × 100 nm).

The preparation procedure of cryo-SPM samples has been reported previously [17]; here, we briefly summarize the key steps in Fig. 1A. A high-purity solution, composed of ultrapure NaCl and water, is loaded into a vessel mounted at the bottom of the frozen-solution preparation apparatus. The solution is purified through repeated freeze–pump–thaw cycles and subsequently frozen to liquid nitrogen (LN_2_) temperature via external cooling (step 1). A clean Au(111) substrate is then brought into contact with the frozen solution, inducing local surface melting and forming a thin interfacial liquid layer (step 2). After sufficient contact, the system is refrozen to a glassy state (step 3), and the substrate is then mechanically detached, transferring a thin layer of frozen NaCl solution onto the Au(111) surface (step 4). Finally, the prepared sample is transferred into the qPlus–AFM system for atomic-resolution imaging.

Due to the cryogenic preparation conditions (LN_2_ temperature), the frozen samples exhibit disordered morphologies with a typical corrugation of ~1.2 nm, which prohibits atomic-scale imaging. To induce crystallization in a controlled manner and meanwhile capture the metastable states, we apply a stepwise low-temperature annealing protocol (140 to 160 K). This treatment enables the formation of hydrated NaCl nanocrystals during the initial crystallization process (Fig. 1B), allowing stable AFM imaging.

**Fig. 2.**
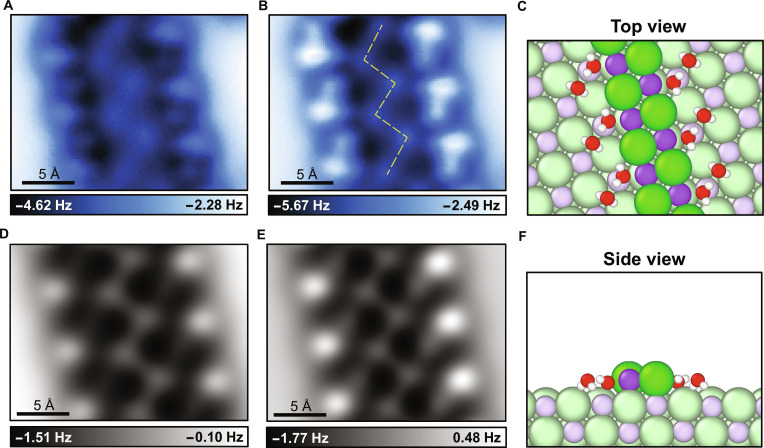
Hydration structure of NaCl zigzag chains. (A and B) Constant-height AFM images of hydrated NaCl nanocrystals in double-stranded chain arrangement at tip heights of −20 and −50 pm. The yellow dashed lines in (B) represent a zigzag pattern composed of chloride ions. (C and D) Corresponding AFM simulation images acquired at tip heights of 8.20 and 7.90 Å. (E and F) Top and side views of the atomic structure of hydrated NaCl nanocrystals. H, O, Na, and Cl atoms are denoted as white, red, purple, and green spheres, respectively.

Atomically resolved AFM images reveal a characteristic zigzag chain-like structure, which is surrounded by dimer-like structures (Fig. 2A and B and Fig. S1). To elaborate the experimental images, we perform detailed AFM simulations based on density functional theory (DFT) calculations. It reveals double-stranded ionic chains, in which sodium and chloride ions alternate with interionic distances matching the lattice constant of bulk NaCl. Chloride ions appear as bright protrusions in constant-height AFM images due to the combined effect of their larger van der Waals radii and the stronger electrostatic repulsion with the negatively charged CO-functionalized tip [18].

Water molecules are observed to adsorb around the chains in dimeric units. Within each dimer, one water molecule adopts an H-down orientation, with both hydrogens pointing toward adjacent chloride ions in the lower layer. The other molecule assumes an H-flat configuration, forming a hydrogen bond with the H-down water molecule. In addition, it also interacts with a sodium ion and a chloride ion of the double-stranded NaCl chain (Fig. 2C and F). The H-down water molecule shows a larger height than the H-flat one, leading to the brighter force contrast in the AFM images.

A well-ordered NaCl lattice is observed beneath the chain-shaped NaCl nanocrystals, indicating homogeneous crystallization progress on the substrate (Fig. S2). The lattice registry with the NaCl substrate is confirmed by the consistent crystallographic orientation of NaCl nanocrystals captured across tens of nanometers (Fig. S3). Similar hydrated NaCl chains, surrounded by water dimer shells, repeatedly appear in different scanning areas, suggesting the universality and robustness of this configuration. The smallest NaCl nanocrystal we found is a zigzag chain consisting of 5 chloride ions and 5 sodium ions hydrated with water dimers (Fig. S4).

**Fig. 3.**
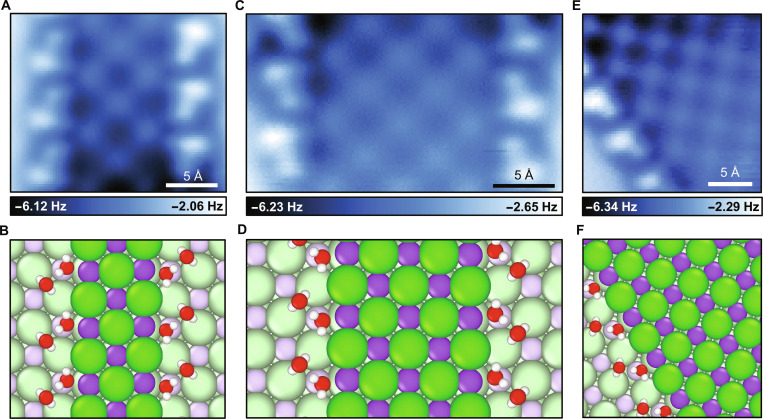
Hydration structures of triple-, 4-, and 5-stranded NaCl nanocrystals. (A and B) Constant-height AFM image and atomic structure of hydrated NaCl nanocrystals in triple-stranded arrangement. (C and D) Constant-height AFM image and atomic structure of hydrated NaCl nanocrystals in 5-stranded arrangement. (E and F) Constant-height AFM image and atomic structure of water dimers at the edge of a large NaCl island. H, O, Na, and Cl atoms are denoted as white, red, purple, and green spheres, respectively, in (B), (D), and (F).

Larger NaCl nanocrystals consisting of triple-stranded (Fig. 3A), 4-stranded (Fig. S5), and 5-stranded ionic chains (Fig. 3C) are also observed. At extended scales, the ionic chains form checkerboard-like patterns. These structures are partially surrounded by water dimers, which do not fully cover the edges of NaCl nanocrystals (Figs. S5 to S7). Occasionally, individual water monomers are also observed (Fig. S4), adsorbed at chain terminals or kink sites where steric constraints prevent the formation of dimers. The water dimers are also observed at the edge of a large crystalline NaCl island (Fig. 3E), suggesting that the water dimers act as the basic units for the hydration of NaCl nanocrystals, instead of water monomers as expected before [19]. DFT calculations further confirm that NaCl clusters hydrated with water dimers are energetically more stable than those with water monomers, as shown in Table S1. We expect that hydration through water dimers should be general for other salts.

**Fig. 4.**
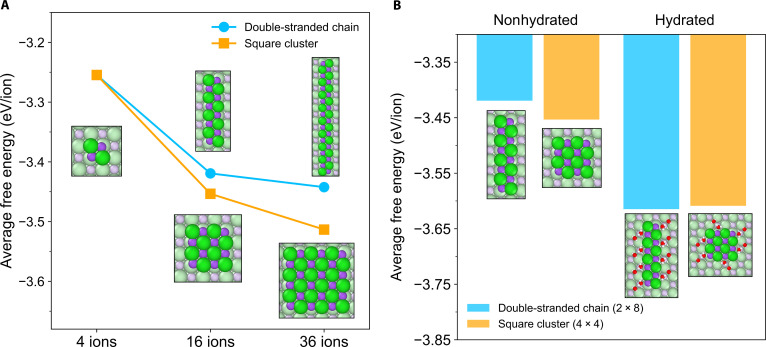
Free energy of different NaCl nanocrystals with and without hydration. (A) Average free energy per ion of isotropic and anisotropic NaCl clusters without water dimers. Lines with blue circles and orange squares represent double-stranded chains and square-shaped clusters, respectively. (B) Comparison of average free energy per ion with and without water dimers. Blue bars and orange bars represent double-stranded chains and square-shaped clusters with 2 × 8 and 4 × 4 shapes, respectively. H, O, Na, and Cl atoms are denoted as white, red, purple, and green spheres, respectively, in the insets in (A) and (B).

The observed structural similarity suggests that water dimers may actively participate in both the initial crystallization process and subsequent crystal growth. DFT calculations support this interpretation, showing that surrounding water dimers stabilize ionic chains by substantially lowering the free energy (Fig. 4B). We propose that this stabilization arises from a specific binding configuration. In each adsorbed water dimer, one water molecule resides within the hydration shell of a sodium ion in the NaCl chain, effectively screening the ionic charge. This water molecule engages in dual interactions: one of its hydrogen atoms forms a hydrogen bond with the other water molecule in the dimer, while the other hydrogen atom forms another hydrogen bond with an adjacent chloride ion. The other water molecule in the dimer orients both of its hydrogen atoms toward chloride ions in the lower layer, anchoring the dimer to the crystal surface. Consequently, the hydrogen-bonding interaction of the dimer is maximized. This dimeric arrangement, acting as a structural anchor through its specific hydrogen-bonding network to multiple ions, collectively stabilizes the entire ionic chain.

For nanocrystals with the same number of ions but different geometries, the number of water dimers adsorbed at the edges can vary, potentially changing their relative stabilities in hydrated versus nonhydrated environments. In the absence of water dimers (Fig. 4A), square-shaped nanocrystals following the isotropic growth pathway are thermodynamically favored because of their more complete ionic bonding. In contrast, when water dimers are present, chain-shaped nanocrystals following the anisotropic growth pathway have a larger perimeter, which allows more water dimers to adsorb along their edges, thereby substantially lowering the overall free energy. For a 16-ion nanocrystal, this additional stabilization makes the anisotropic, chain-shaped configuration energetically more favorable (Fig. 4B), explaining its frequent occurrence during the early stages of crystal growth.

As the nanocrystals grow larger, edge effects diminish and bulk ionic interactions dominate, enabling the intrinsic free-energy advantage of the isotropic, square-shaped structures to prevail and ultimately driving the formation of crystalline NaCl. Thus, the water dimers transiently tip the thermodynamic balance toward anisotropic growth in NaCl nanocrystals, but isotropic growth regains dominance as the size increases.

## Conclusion

In summary, we employ a cryo-SPM approach to directly visualize hydrated NaCl nanocrystals at atomic resolution. To the best of our knowledge, this represents the first experimental real-space imaging of an anisotropic growth pathway during the early stages of NaCl crystallization. We observe double- to 5-stranded ionic chains, stabilized by surrounding water dimers that lower the free energy of NaCl nanocrystals and promote anisotropic growth at the nanoscale. DFT calculations further confirm that the water dimer adsorption provides substantial stabilization to chain-shaped configurations. Together, these findings underscore the critical role of water dimers in the initial crystallization process of salt and demonstrate the potential of cryo-SPM as a powerful technique for elucidating crystallization pathways, particularly in hydrated environments. These results suggest that controlling the local hydrogen-bonding environment may offer a practical route to tuning crystallization morphology in related ionic materials.

## Methods

### Sample preparation

Sodium chloride (NaCl, from Sigma-Aldrich, 99.9%) and deuterium oxide (D_2_O, from Sigma-Aldrich, 99.9%) were sequentially added into the reagent vessel, and dissolution was promoted via ultrasonication. The vessel was mounted onto the gate valve at the bottom of the frozen-solution sample preparation apparatus. The solution in the vessel was further purified under vacuum by 3 to 5 freeze–pump–thaw cycles to remove remaining gas impurities. The Au(111) single crystals used as substrate was purchased from MaTeck. The Au(111) crystal was cleaned by repeated Ar^+^ ion sputtering at 1 keV and annealing at about 700 K for multiple cycles.

The preparation procedure of the frozen-solution sample followed the method described in our previous publication [17]. After preparation, the sample was promptly transferred to the preparation chamber. Annealing was performed on the manipulator at 140 to 160 K. The temperature was monitored by a DT670 silicon diode (Lakeshore) installed close to the sample stage. Using our custom-developed temperature control system, we achieved a standard deviation at the set-point temperature within 0.5 K [20]. All as-grown and annealed samples were quickly transferred to an AFM scanner kept at 4.7 K for further AFM measurements, by using a wobble stick precooled by a 77-K stage on the outer shield for over 1 h.

### AFM measurements

All experiments were performed at 5 K with a combined non-contact AFM and scanning tunneling microscopy system, by using a homemade qPlus sensor equipped with a tungsten tip (spring constant, *k*_0_ ≈ 1,800 N m^−1^; resonance frequency, *f*_0_ = 27.8 kHz; quality factor, *Q* ≈ 50,000). All AFM data were measured at 4.7 K under ultrahigh vacuum (<2 × 10^−10^ mbar). All of the AFM topographic images and the AFM frequency-shift (Δ*f*) images were obtained with the CO-functionalized tips in constant-frequency-shift or constant-height modes, respectively. The oscillation amplitude of AFM imaging was 100 pm if not specifically mentioned. Only the relative heights between images had a certain reference value. Image processing was performed by Gwyddion [21].

### DFT calculations

DFT calculations were performed using the Vienna Ab initio Simulation Package (VASP version 6.1) [22,23]. Projector-augmented wave pseudopotentials were used with a cutoff energy of 500 eV for the expansion of the electronic wave functions[24]. In the DFT calculations, the system consisted of NaCl nanocrystals with surrounding water molecules on top of a NaCl (001) substrate modeled by a double-layer slab. The lattice constant of the NaCl unit cell was set to be 5.692 Å, and both the 2 atomic layers of the NaCl substrate were unfixed in the DFT calculations. Monkhorst–Pack *k*-point meshes of spacing denser than 2*π* × 0.04 Å^−1^ were used, and the thickness of the vacuum slab was larger than 15 Å. The geometry optimizations were performed with an energy criterion of 0.0001 eV and a force criterion of 0.05 eV/Å.

### Simulation of AFM images

The ∆f images were simulated with a molecular mechanics model, based on methods described previously [25]. We used the following parameters for the flexible probe-particle tip model: effective lateral stiffness $k=0.25\;N\;m^{-1}$ and effective atomic radius $R_{c}=1.661\;Å$. A quadrupole-like charge distribution at the tip apex was used to simulate the CO tip with q=−0.10e (e is the elementary charge, and q is the magnitude of quadrupole charge at the tip apex). The electrostatic potential used in AFM simulations were obtained from DFT calculations. The parameters r (van der Waals radius) and ε (potential well depth) of the Lennard-Jones pairwise potentials for the O and H atoms used in AFM simulations are $r_{H}=1.487\;Å$, $\varepsilon_{H}=0.681\;\mathrm{meV}$, $r_{O}=2.39\;Å$, and $\varepsilon_{O}=9.11\;\mathrm{meV}$, while those of Na^+^ and Cl^−^ ions are $r_{{\mathrm{Na}}^{+}}=1.40\;Å$, $\varepsilon_{{\mathrm{Na}}^{+}}=15.32\;\mathrm{meV}$, $r_{{\mathrm{Cl}}^{-}}=1.95\;Å$, and $\varepsilon_{{\mathrm{Cl}}^{-}}=0.56\;\mathrm{meV}$. Such selection of values integrates parameters from previous studies [16] and fitting of experimentally obtained force spectra. The tip height in the AFM simulations is defined as the vertical distance between the metal tip apex and the topmost layer of the substrate. The oscillation amplitudes of AFM tip during the simulation were 100 pm.

### DFT-calculated average free energy per ion in different NaCl clusters

The software and the parameter settings were consistent with the above section. To explain the definition of average free energy in Fig. 4, we can represent the process of the clusters forming on the surface using the following imaginary reaction equation:

$$n{\mathrm{Na}}^{+}\left(\mathrm{aq}\right)+n{\mathrm{Cl}}^{-}\left(\mathrm{aq}\right)+\left(\mathrm{sub}.\right)\left(s\right)\to \left(\mathrm{sub}.\right)\cdot {\mathrm{Na}}_{n}{\mathrm{Cl}}_{n}\left(s\right)$$ (1)

in which $\left(\mathrm{sub}.\right)$ represents the chemical formula of the substrate, while $\left(\mathrm{sub}.\right)\cdot {\mathrm{Na}}_{n}{\mathrm{Cl}}_{n}$ represents the whole system with cluster ${\mathrm{Na}}_{n}{\mathrm{Cl}}_{n}$ adsorbed on the substrate. The above reaction equation can be broken down into 2 subreactions:

$$n{\mathrm{Na}}^{+}\left(\mathrm{aq}\right)+n{\mathrm{Cl}}^{-}\left(\mathrm{aq}\right)\to {\mathrm{Na}}_{n}{\mathrm{Cl}}_{n}\left(\mathrm{aq}\right)$$ (2)

$${\mathrm{Na}}_{n}{\mathrm{Cl}}_{n}\left(\mathrm{aq}\right)+\left(\mathrm{sub}.\right)\left(s\right)\to \left(\mathrm{sub}.\right)\cdot {\mathrm{Na}}_{n}{\mathrm{Cl}}_{n}\left(s\right)$$ (3)

The free energy of a NaCl cluster on the substrate contains 3 parts: (a) the free energy of formation of all ions $n{∆}_{f}G_{{\mathrm{Na}}^{+}}\left(\mathrm{aq}\right)+n{∆}_{f}G_{{\mathrm{Cl}}^{-}}\left(\mathrm{aq}\right)$; (b) the interaction energy between ions within the cluster, that is, the reaction free energy for forming the cluster $\begin{array}{c} {∆}_{r}G_{1}={∆}_{f}G_{{\mathrm{Na}}_{n}{\mathrm{Cl}}_{n}}\left(\;\mathrm{aq}\;\right)-n{∆}_{f}G_{{\mathrm{Na}}^{+}}\left(\mathrm{aq}\right)- \end{array}$$\begin{array}{c} n{∆}_{f}G_{{\mathrm{Cl}}^{-}}\left(\mathrm{aq}\right) \end{array}$; and (c) the interaction energy between the cluster and the substrate, namely, the reaction free energy for the second subreaction $\begin{array}{c} {∆}_{r}G_{2}={∆}_{f}G_{\left(\mathrm{sub}.\right)\cdot {\mathrm{Na}}_{n}{\mathrm{Cl}}_{n}}\left(s\right)-{∆}_{f}G_{\left(\mathrm{sub}.\right)}\left(s\right)- \end{array}$$\begin{array}{c} {∆}_{f}G_{{\mathrm{Na}}_{n}{\mathrm{Cl}}_{n}}\left(\mathrm{aq}\right) \end{array}$. Therefore, the average free energy per ion in the NaCl cluster can be calculated using the following expression:

$$\begin{array}{c} ∆G_{\mathrm{ave}}=\frac{n{∆}_{f}G_{{\mathrm{Na}}^{+}}\left(\mathrm{aq}\right)+n{∆}_{f}G_{{\mathrm{Cl}}^{-}}\left(\mathrm{aq}\right)+{∆}_{r}G_{1}+{∆}_{r}G_{2}}{2n}\\ =\frac{{∆}_{f}G_{\left(\mathrm{sub}.\right)\cdot {\mathrm{Na}}_{n}{\mathrm{Cl}}_{n}}\left(s\right)-{∆}_{f}G_{\left(\mathrm{sub}.\right)}\left(s\right)}{2n} \end{array}$$ (4)

where ${∆}_{f}G_{\left(\mathrm{sub}.\right)\cdot {\mathrm{Na}}_{n}{\mathrm{Cl}}_{n}}\left(s\right)$ and ${∆}_{f}G_{\left(\mathrm{sub}.\right)}\left(s\right)$ represent the free energies of the whole system and the substrate, respectively. For each structure, geometry optimizations were performed with an energy criterion of 0.0001 eV and a force criterion of 0.05 eV/Å.

Similarly, for the hydrated cluster, the average free energy per ion can be calculated using the following expression:

$$∆G_{\mathrm{ave}}=\frac{{∆}_{f}G_{\left(\mathrm{sub}.\right)\cdot {\mathrm{Na}}_{n}{\mathrm{Cl}}_{n}\cdot mH_{2}O}\left(s\right)-{∆}_{f}G_{\left(\mathrm{sub}.\right)\cdot mH_{2}O}\left(s\right)}{2n}$$ (5)

where ${∆}_{f}G_{\left(\mathrm{sub}.\right)\cdot mH_{2}O}\left(s\right)$ represents the free energy of the system with m water molecules adsorbed on the substrate.

## Data Availability

The data used to create the figures in the main text of this study are available at Zenodo (https://doi.org/10.5281/zenodo.17699313) [26]. All other data needed to evaluate the conclusions in the paper are present in the paper or Supplementary Materials.
