# Supplementary material for: The Role of Water Dimers in the Initial Stage of Salt Crystallization
Source: Research (Wash D C). 2025 Dec 23;8:1040. doi: 10.34133/research.1040 (PMC12722634; doi:10.34133/research.1040)
Supplement: Supplementary 1 — Figs. S1 to S7 Table S1 [file research.1040.f1.pdf]

Supplementary Information

The Role of Water Dimers in the Initial Stage of Salt Crystallization

Jiadong Guo†, Xinmeng Liu†, Yunzhe Jia†, Junhao Xie, Yuejian Zhang, Yipeng He, Jiyu Xu, Cui Zhang, Duanyun Cao, Sheng Meng*, and Ying Jiang*

† These authors contributed equally to this work.

*Address correspondence to: Sheng Meng; smeng@iphy.ac.cn, and Ying Jiang; yjiang@pku.edu.cn

Contents:

Supplementary Table 1

Supplementary Figures 1-7

Supplementary Table

Table S1: Calculated average free energies (E_{ave}) of the two NaCl nanocrystal configurations shown in Fig. 4b under different hydration conditions. The values are obtained from DFT calculations considering three scenarios: hydration by water dimers, hydration by water monomers, and the absence of hydration.

**NaCl nanocrystal shapes**	**E_{ave} with water dimers / eV**	**E_{ave} with water monomers /eV**	**E_{ave} without water / eV**
Double-stranded chain (2×8)	-3.615	-3.558	-3.419
Square cluster (4×4)	-3.609	-3.562	-3.453

Supplementary Figures

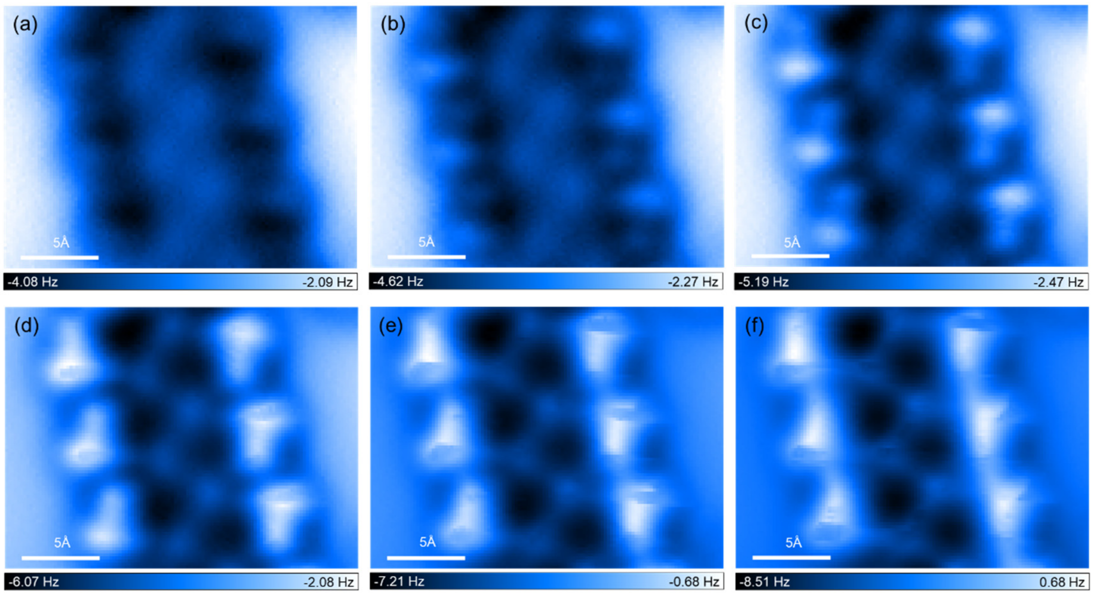

Fig. S1. Detailed AFM characterization of a hydrated NaCl nanocrystal in double-stranded arrangement. (a)-(f) Constant-height AFM images of the nanocrystal shown in Fig. 2, with the tip heights of 0 pm (a), -20 pm (b), -40 pm (c), -60 pm (d) , -80 pm (e) and -100 pm (f).

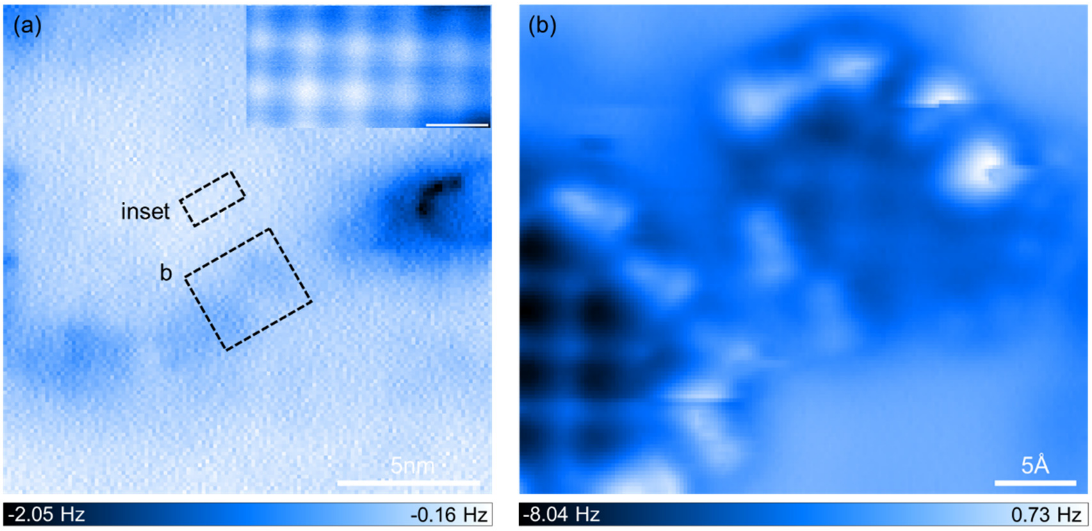

Fig. S2. Hydrated nanocrystals formed on the crystalline NaCl surface. (a) Constant-height AFM images of a selected area. Size: 17 nm × 17 nm. The black dashed rectangle outlines the hydrated nanocrystals in (b) and the underlying crystalline NaCl lattice (inset). (b) Constant-height AFM image of the hydrated nanocrystals. Tip heights: 0 pm (a), -890 pm (inset), and -480 pm (b).

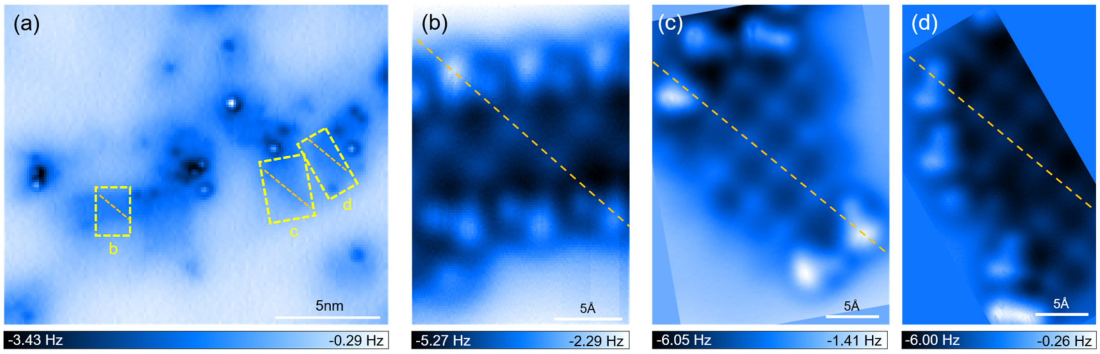

Fig. S3. Hydrated NaCl nanocrystals with the same orientation formed in the same area. (a) Constant-height AFM images of a selected area. Size: 15 nm × 18 nm. (b)-(d) Constant-height AFM images of different nanocrystals shown by the yellow dashed rectangular boxes in (a). The rotation angles relative to (a) are 0°(b), 10°(c), and 30°(d), respectively. The yellow orthogonal arrows indicate the consistent lattice orientation.

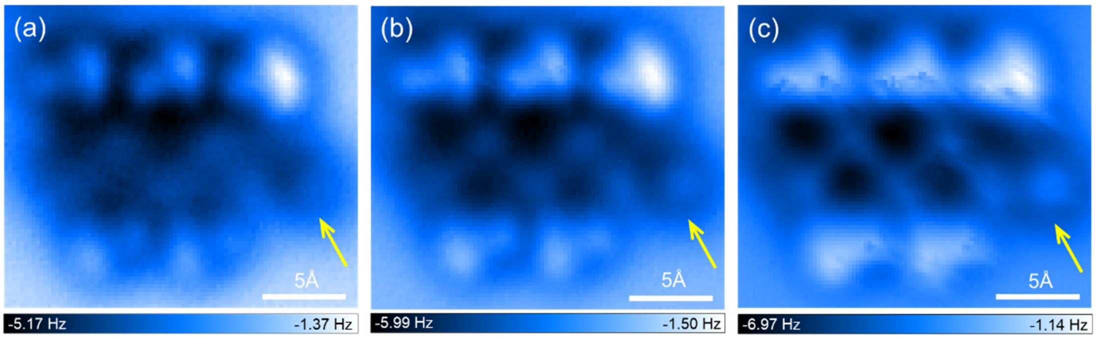

Fig. S4. Detailed AFM characterization of a small hydrated NaCl nanocrystal in double-stranded arrangement. (a)-(c) Constant-height AFM images of the nanocrystal, with the tip heights of 0 pm (a), -20 pm (b), -40 pm (c). The yellow arrows indicate a water monomer.

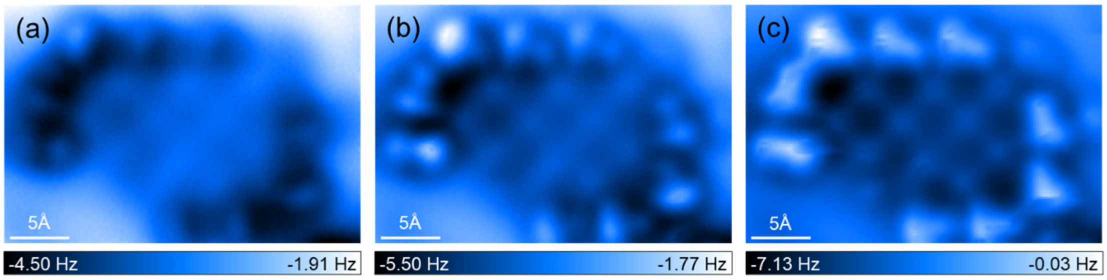

Fig. S5. Detailed AFM characterization of the four-stranded NaCl chain. Constant-height AFM images with the tip heights of 0 pm (a), -50 pm (b) and -100 pm (c).

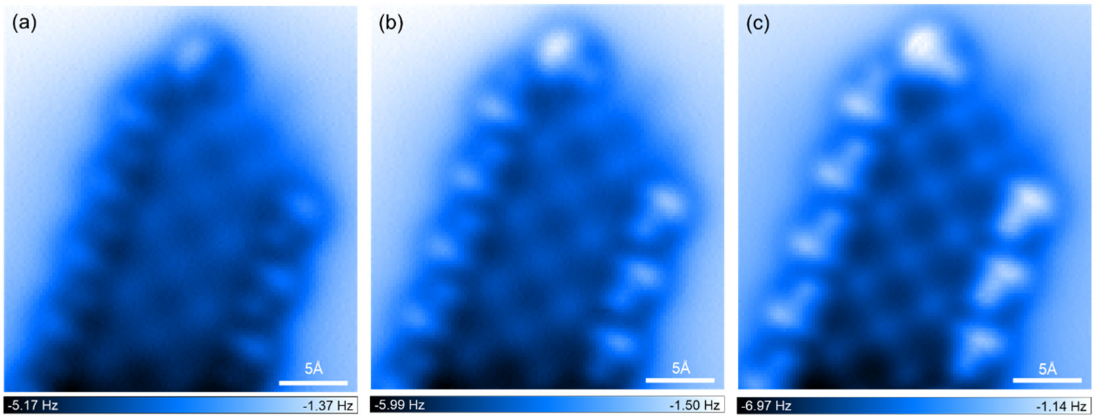

Fig. S6. Detailed AFM characterization of a triple-stranded NaCl chain. (a)-(c) Constant-height AFM images of the triple-stranded ionic chain, with the tip heights of 0 pm (a), -20 pm (b) and -40 pm (c).

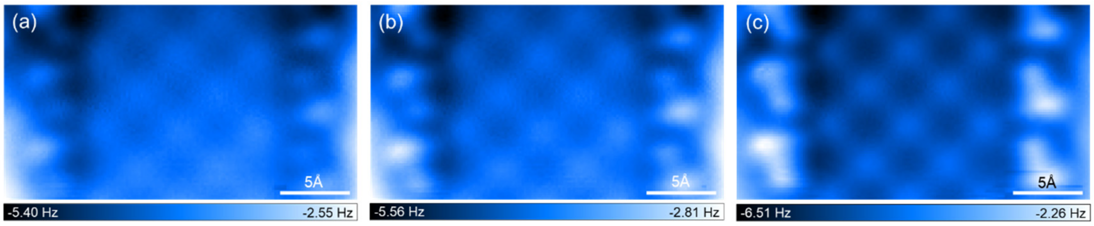

Fig. S7. Detailed AFM characterization of a five-stranded NaCl chain. (a)-(c) Constant-height AFM images of the five-stranded ionic chain, with the tip heights of 0 pm (a), -10 pm (b) and -30 pm (c).
